# HOW DO PLACES OF RESIDENCE OVER THE LIFE COURSE SHAPE THE DYNAMICS OF HEALTH DISPARITIES IN LATER LIFE?

**DOI:** 10.1093/geroni/igae098.0693

**Published:** 2024-12-31

**Authors:** Zhuoer Lin, Xi Chen

**Affiliations:** University of Illinois Chicago, Chicago, Illinois, United States; Yale University, New Haven, Connecticut, United States

## Abstract

**Background:**

Significant geographical disparities persist in cognitive and health outcomes. According to life course theory, these disparities may stem from a history of place-based exposures throughout lifespan. However, due to data limitations, no study has explored how residential history impacts later-life health inequalities.

**Methods:**

We addressed this gap by constructing an exceptionally comprehensive residential history dataset. Drawing from the Health and Retirement Study, we identified state-level places of residence for each individual from birth to age 60, and subsequently linked this information to a broad range of cognitive and health outcomes in later life (N=10,124). Employing regression-based Shapley value decomposition, we quantified the contribution of place of residence at each age to cognitive and health outcomes, thereby delineating the trajectories of place effects on cognition and health.

**Results:**

Our analysis revealed distinct contributions of place of residence at various ages, particularly in relation to cognitive health compared to other health outcomes. Notably, place of residence exerted the most significant influence on cognition at birth, with this contribution decreasing linearly and rapidly during the initial 20 years of life (from age 0 to age 20), before stabilizing during adulthood. In contrast, we observed an overall upward trend in the contribution of place of residence to other health outcomes such as disability and depression.

**Discussion:**

Our findings underscore the dynamic nature of the contribution of place of residence to health inequalities over the life course, emphasizing substantial disparities in the effects of location on cognitive health compared to other health outcomes.

